# Molecular epidemiology of Japanese encephalitis virus circulating in South Korea, 1983-2005

**DOI:** 10.1186/1743-422X-7-127

**Published:** 2010-06-14

**Authors:** Seok-Min Yun, Jung Eun Cho, Young-Ran Ju, Su Yeon Kim, Jungsang Ryou, Myung Guk Han, Woo-Young Choi, Young Eui Jeong

**Affiliations:** 1 WHO Japanese Encephalitis Regional Reference Laboratory for the Western Pacific Region/Division of Arboviruses, National Institute of Health, Korea Centers for Disease Control and Prevention, Seoul, Republic of Korea

## Abstract

We sequenced the envelope (E) gene of 17 strains of the Japanese encephalitis virus (JEV) isolated in South Korea in 1983-2005 and compared the sequences with those from previously reported strains. Our results show the remarkable genetic stability of the E gene sequence in Korean JEV strains. Five pairs of E gene sequences from 10 Korean strains were identical, despite geographical differences and a maximum five-year time span. Sequence comparisons with other Asian strains revealed that the Korean strains are closely related to those from China, Japan, and Vietnam. Genotype 3 strains were predominant in Korea before 1993, when genotype 1 strain K93A07 was first isolated. The two genotypes were detected simultaneously in 1994 but since then, only genotype 1 has been isolated in South Korea. Thus, the genotype change occurred according to the year of isolation rather than the geographical origin.

## Findings

Japanese encephalitis virus (JEV) is a mosquito-borne flavivirus (genus *Flavivirus*, family *Flaviviridae*), which causes acute viral encephalitis in humans. Approximately 30,000-50,000 cases, with 10,000 deaths, are reported annually throughout Asia [1]. The JEV genome is a positive-sense, single-stranded RNA molecule, approximately 11 kb in length. The polyprotein is processed into three structural proteins, the capsid (C), membrane (M), and envelope (E) proteins, and seven nonstructural proteins, NS1, NS2A, NS2B, NS3, NS4A, NS4B, and NS5 [2].

Generally, RNA viruses have intrinsically high mutation rates and consequently greater potential for rapid evolution than the DNA viruses [3]. Many studies have revealed the phylogenetic relationships among the JEV strains. Although full-genome sequences provide the most reliable information, it takes several weeks to fully sequence a strain and an enormous computing capacity is required for the analysis of large sequences. Therefore, much shorter sequences from various genes are typically evaluated as phylogenetic markers. Historically, 3-4 JEV genotypes have been proposed based on short sequences (198 nt, 240 nt, or 280 nt) in the C/prM region [4-6], but such short sequences are insufficient to identify exact relationships. Therefore, the complete E gene (1,500 nt) is preferred as a marker and 4-5 genotypes have been reported in phylogenetic analyses [7-10]. To date, the molecular epidemiology of JEV strains has been well studied in Asian countries, including China, Japan, India, Taiwan, Thailand, and Vietnam [6,11-14]. However, the molecular characterization of the Korean strains, including their genetic diversity, has not been well documented. Although over 100 JEV strains have been isolated during extensive mosquito surveillance since 1975, most of them have been lost, without further study. To date, only three strains have been fully sequenced: K87P39, K94P05, and KV1899 [15-17]. However, the C/prM or E genes of other strains, such as K82P01, K91P55, and K93P05, have been sequenced [18,19].

Previous studies have only dealt with a few Korean strains isolated before 1999, and more recent strains must be analyzed to fully characterize the molecular epidemiology of JEV in South Korea. In this study, we sequenced the complete E genes of 17 Korean JEV strains isolated between 1983 and 2005 and analyzed their genetic variation and their relationships to other Asian strains.

**Figure 1:**
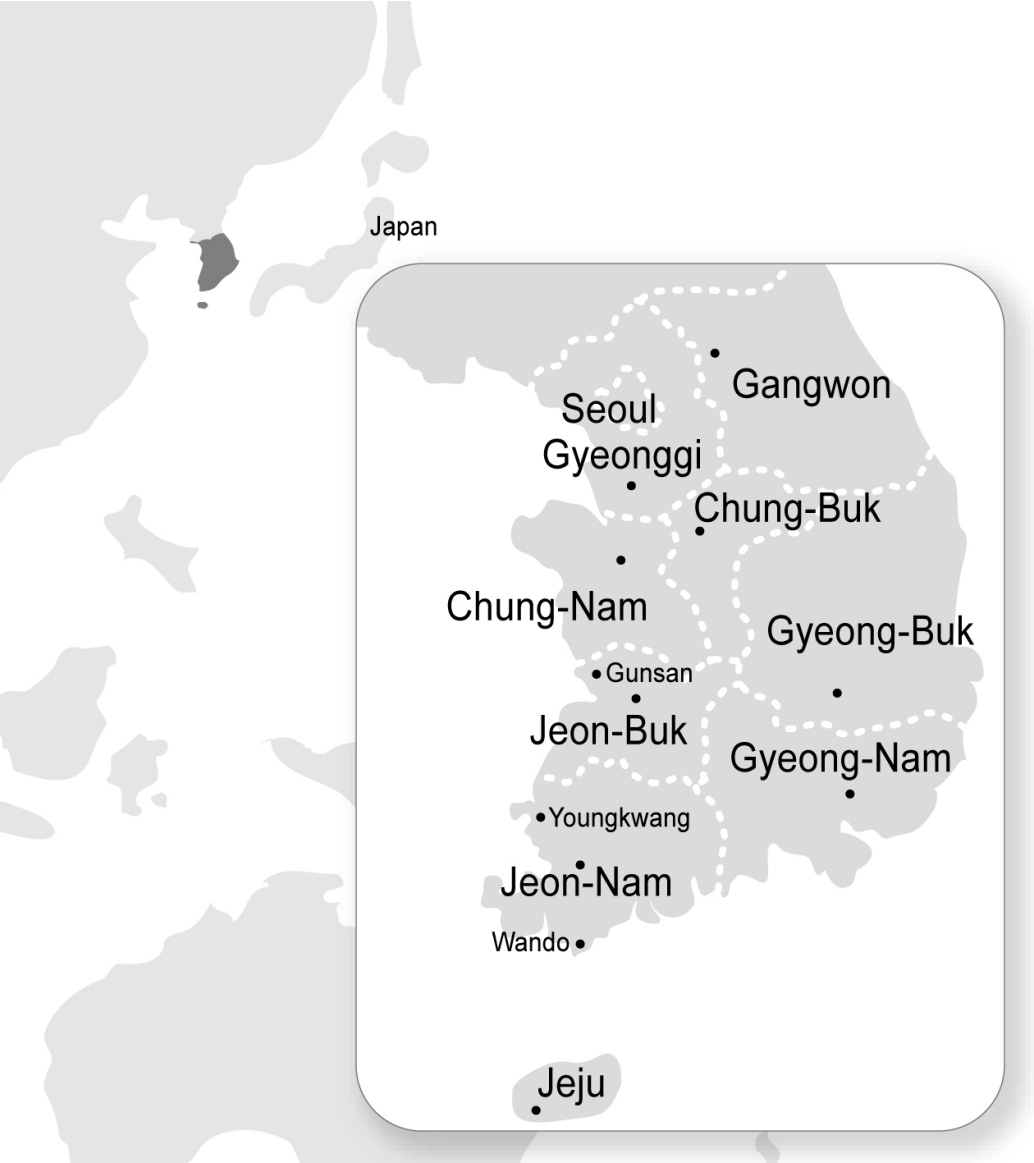
**Locations of JEV vector surveillance in South Korea**. Mosquitoes were caught in nine provinces, excluding Seoul, once a week between July and September. The mosquito collection sites are indicated as closed circles. Youngkwang and Wando are located in Jeon-Nam Province. Gunsan is located in Jeon-Buk Province.

Since 1975, the Korea National Institute of Health has annually checked JEV activity from vector mosquitoes collected between July and September in nine provinces of South Korea (Figure 1). Black-light traps were operated once a week in cattle sheds and the mosquitoes were identified morphologically and categorized to the species level. Only *Culex tritaeniorhynchus *mosquitoes (the major JEV vector in Korea) were processed for virus isolation, using suckling mice as described previously [18]. Seventeen strains from among the JEV strains isolated in South Korea in 1983-2005 were initially characterized in the present study (Table 1). Viral RNA was extracted from the stocks of each virus using the QIAamp Viral RNA Mini Kit (Qiagen, Valencia, CA, USA). The purified RNA was used as the template for cDNA synthesis using the SuperScript™ III first-strand synthesis system (Invitrogen, Carlsbad, CA, USA) with primer JE-2623AS (NS1 region, 5'-GCTTTGTGGACGATCTTCGC-3'), according to the manufacturer's instructions. The synthesized cDNA was then used for PCR amplification with AccuPrime™ *Pfx *DNA polymerase (Invitrogen) and primers JE-723 S (prM/M region, 5'-CGGACCAGGCATTCCAA-3') and JE-2623AS. The primers were designed according to the consensus sequences of three Korean JEV strains (K94P05, K87P39, and KV1899). The amplified products (1.9 kb) were purified and sequenced using the ABI PRISM BigDye Terminator Cycle Sequencing Kit and an ABI 3730 × l sequencer (Applied Biosystems, Foster City, CA, USA) at Macrogen (Seoul, Korea). The nucleotide sequences of the E genes (1,500 nt) were compared with those of other JEV strains representing each genotype and different geographic regions. A total of 86 E gene sequences were initially collected and 29 strains representing each country and genotype were finally selected (Table 2). A multiple alignment was generated with the ClustalX 2.0.11 program [20] and the percentage similarities between the aligned sequences were calculated using the MegAlign program implemented in the Lasergene software (DNASTAR, Madison, WI, USA). Phylogenetic analyses based on the E gene were performed with the neighbor-joining (NJ) and maximum likelihood (ML) methods using MEGA 4.0 [21] and TREE-PUZZLE 5.2 [22], respectively. The E gene sequence of the Murray Valley encephalitis virus (MVEV) was used as the outgroup (GenBank accession no. NC_000943). For the NJ tree, the Tamura-Nei model was used to compute the genetic distances, and the reliability of the tree was tested by bootstrap analysis with 1,000 replications. For the ML tree, the HKY85 evolutionary model of nucleotide substitution was used to build the tree based on the complete E gene. The statistical significance of each internal branch of the tree was indicated as a quartet puzzling (QP) value. Other parameters for the ML tree are available upon request. All the trees were produced with the MEGA 4.0 software.

**Table 1:** Details of 22 strains of JEV from South Korea*

Strain	Year	Source	Location	**Accession no**.
**K82P01**	1982	*Culex tritaeniorhynchus*	Youngkwang	U34926
K83P34	1983	*Culex tritaeniorhynchus*	IU	FJ938231
K83P44	1983	*Culex tritaeniorhynchus*	IU	FJ938232
K84A071	1984	*Culex tritaeniorhynchus*	IU	FJ938224
K87A07	1987	*Culex tritaeniorhynchus*	IU	FJ938225
K87A071	1987	*Culex tritaeniorhynchus*	IU	FJ938226
**K87P39**	1987	*Culex tritaeniorhynchus*	Wando	U34927
K88A07	1988	*Culex tritaeniorhynchus*	IU	FJ938227
K88A071	1988	*Culex tritaeniorhynchus*	IU	FJ938228
K89A07	1989	*Culex tritaeniorhynchus*	IU	FJ938229
**K91P55**	1991	*Culex tritaeniorhynchus*	Wando	U34928
K93A07	1993	*Culex tritaeniorhynchus*	IU	FJ938230
K94A07	1994	*Culex tritaeniorhynchus*	IU	FJ938216
K94A071	1994	*Culex tritaeniorhynchus*	IU	FJ938217
**K94P05**	1994	*Culex tritaeniorhynchus*	Wando	U34929
K95A07	1995	*Culex tritaeniorhynchus*	IU	FJ938218
K96A07	1996	*Culex tritaeniorhynchus*	IU	FJ938219
**KV1899**	1999	Pig serum	Gyeonggi	AY316157
K01-GN	2001	*Culex tritaeniorhynchus*	Gyeong-Nam	FJ938220
K01-JB	2001	*Culex tritaeniorhynchus*	Jeon-Buk	FJ938221
K01-JN	2001	*Culex tritaeniorhynchus*	Jeon-Nam	FJ938222
K05-GS	2005	*Culex tritaeniorhynchus*	Gunsan	FJ938223

* Five isolates sequenced previously are indicated in boldface type.

IU: information unavailable.

**Table 2:** Details of 29 JEV strains compared with Korean strains

Strain	Year	Location	Source	Genotype	**Accession no**.
Fu	1995	Australia	Human serum	2	AF217620
P3	1949	China	Human brain	3	AY243844
YN	1954	China	Human brain	3	AY243838
SA 14	1954	China	Mosquito	3	U14163
YN79-Bao83	1979	China	Mosquito	1	DQ404128
YN86-B8639	1986	China	Mosquito	1	DQ404133
SH-53	2001	China	Mosquito	1	AY555757
SH03-124	2003	China	Mosquito	1	DQ404100
SH04-3	2004	China	Mosquito	3	DQ404105
SH17M-07	2007	China	Mosquito	1	EU429297
GP78	1978	India	Human brain	3	AF075723
JKT5441	1981	Indonesia	Mosquito	2	U70406
JKT7003	1981	Indonesia	Mosquito	4	U70408
JKT9092	1981	Indonesia	Mosquito	4	U70409
Nakayama	1935	Japan	Human brain	3	U70413
JaOH0566	1966	Japan	Human brain	3	AY029207
JaOArS1186	1986	Japan	Mosquito	3	AB028262
JaOArK5990	1990	Japan	Unknown	3	AB028268
Ishikawa	1994	Japan	Pig	1	AB051292
JaNAr0990	1990	Japan	Mosquito	3	AY427797
JaNAr32-04	2004	Japan	Mosquito	1	FJ185151
PhAn1242	1984	Philippines	Pig serum	3	U70417
B1065	1983	Thailand	Pig blood	2	U70388
ThCMAr6793	1993	Thailand	Mosquito	1	D45363
H49778	1987	Sri Lanka	Human brain	3	U70395
VN207	1986	Vietnam	Human brain	3	AY376461
VN50	1989	Vietnam	Human brain	3	AY376463
VN78	2002	Vietnam	Mosquito	1	AY376467
Muar	1952	Singapore	Human brain	5	[30]

**Figure 2:**
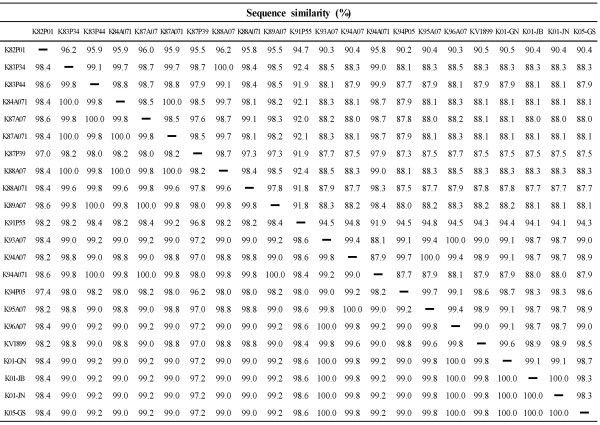
**Nucleotide and amino acid sequence similarities among Korean JEV strains**. The percentage similarities between the aligned nucleotide and deduced amino acid sequences were calculated (uncorrected p-distances) with the MegAlign program implemented in the Lasergene software. The nucleotide similarities (%) are shown above the diagonal and the deduced amino acid identities (%) are shown below the diagonal.

Twenty-two Korean JEV strains showed minimal sequence similarities (uncorrected p-distances) of 87.3% and 96.2% at the nucleotide and amino acid sequence levels, respectively (Figure 2). Except for K82P01 and K91P55 strains, Korean JEV strains were divided into two groups, genotypes 1 and 3. The nucleotide sequence divergence within the genotypes were only 0.3-1.7% (mean 1.1%, genotype 1) and 0.1-2.7% (mean 1.5%, genotype 3), respectively. The sequence divergence between the two genotypes was 11.5%-12.7% (mean 12.0%). K82P01 showed nucleotide divergences of 9.5%-9.8% from the genotype 1 strains and 3.8%-4.5% from the genotype 3 strains. K91P55 showed nucleotide divergences of 5.2%-5.9% from genotype 1 and 7.6-8.2% from genotype 3.

**Figure 3:**
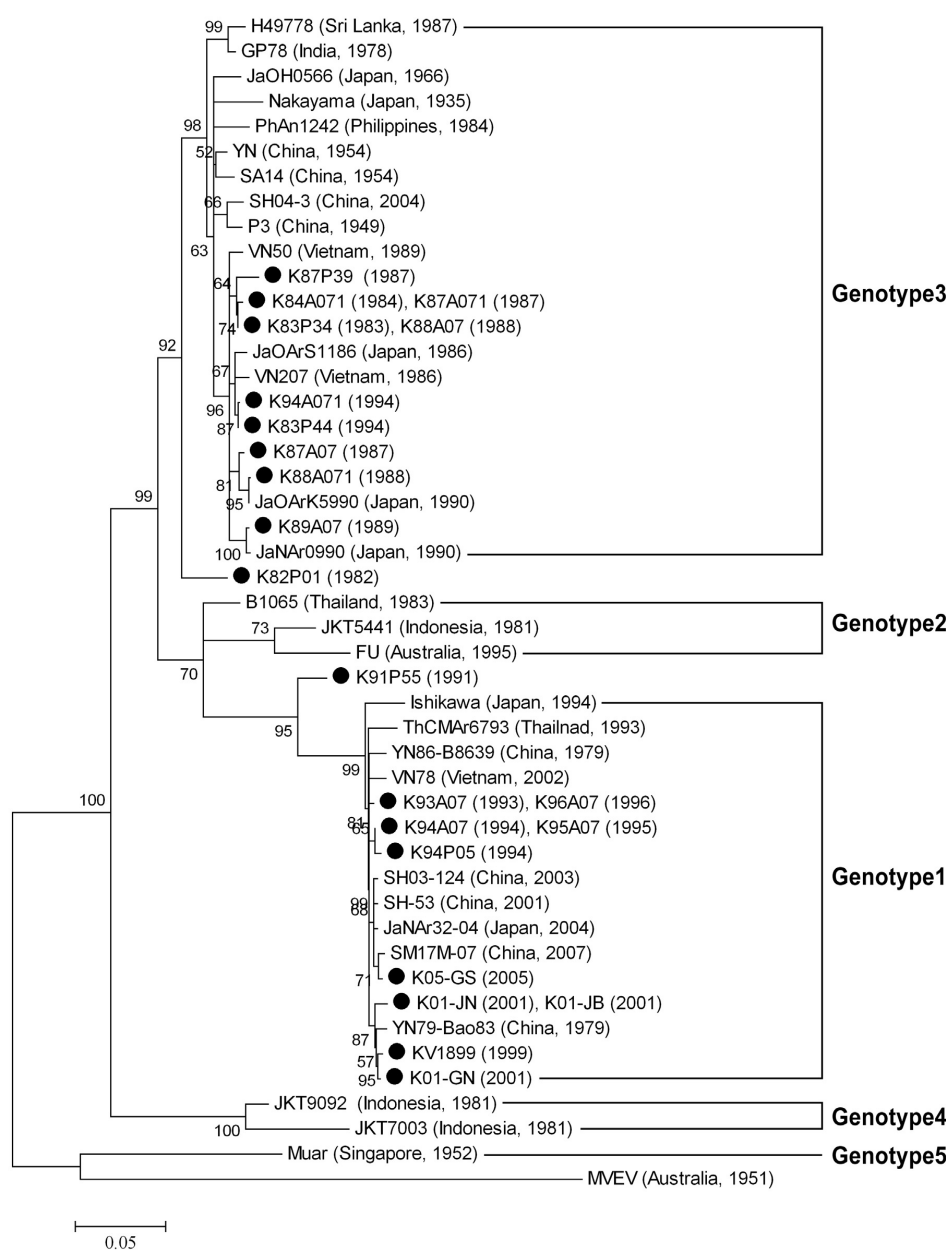
**Maximum likelihood tree of 51 JEV strains representing four different genotypes, including 22 Korean strains**. The HKY85 evolutionary model of nucleotide substitution was used to construct a ML tree for the complete E gene sequence. The tree was rooted with the E gene sequence of the Murray Valley encephalitis virus (MVEV, accession no. NC_000943). Branch reliability is indicated with quartet puzzling (QP) values. Branches showing QP reliability > 70% can be considered well supported [22]. The scale bar indicates the number of base substitutions per site. Korean strains are indicated as closed circles and the JEV genotypes are as defined previously [8].

The E gene sequences of the Korean JEV strains showed remarkable genetic stability. Five pairs of E gene sequences from 10 Korean strains (K83P34 and K88A07, K84A071 and K87A071, K93A07 and K96A07, K94A07 and K95A07, and K01JN and K01-JB) were identical, despite differences in their geographic distributions and the maximum five-year time span. This genetic stability in JEV was also detected in strains from Taiwan, China, and Japan [6,13,23]. When phylogenetic analyses were performed, the branching patterns on both the NJ and ML trees were similar, with slight differences in the reliability indices. Thus, only ML tree is presented in this study (Figure 3). Most Korean strains were divided into genotypes 1 and 3. Ten Korean strains grouped in genotype 3, together with those isolated in China, Japan, India, Philippines, Sri Lanka, and Vietnam between the 1930 s and the early 1990 s. Another 10 Korean strains clustered in genotype 1, together with strains isolated in China, Japan, Thailand, and Vietnam between the late 1970 s and the present day. Historically, the genotypic classification of some JEV strains was discordant, depending on the phylogenetic markers or tree construction method used, including substitution models.

Two Korean JEV strains, K82P01 and K91P55, were notably problematic in phylogenetic analyses. K82P01 was grouped in genotype 3 or unclassified based on the E gene sequence, and K91P55 was classified in either genotype 1 or genotype 3 depending on the gene region used for the phylogenetic analysis [7,10,16]. Moreover, in an analysis of *Flavivirus *recombination, K82P01 and K91P55 appeared to be putative recombinant strains derived from genotype 1 and 3 strains [24]. When we analyzed the two strains using the RDP3 program [25], both were shown to be recombinant strains (data not shown). Chuang and Chen recently provided experimental evidence that RNA recombination occurs in JEV [26]. This genotypic conflict may be confirmed by sequencing the E gene again or, more effectively, the full genome.

However, in this study, we could not pursue this research because the two strains were lost during long-term storage. Therefore, we suggest that these two strains are not used in future studies of JEV evolution. Our results indicate that the genotypes of the Korean JEV strains changed from genotype 3 to genotype 1 around 1993, with both genotypes isolated in 1994 (Figure 3). Since then, only genotype 1 strains have been isolated in South Korea. Before the present study, it was reported that genotype 1 was introduced into Korea around 1991 (K91P55 strain) or 1994 (K94P05 strain) [14,15,27].

Interestingly, this genotype change was also reported in Japan in 1991 [12,23], in China in 1979 [13], in Vietnam in 2001 [9], and in Thailand in 1991 [14]. Although several explanations have been offered [5,7,9], we believe that migrating water birds may be a major mediator of the new genotypes in these regions. Consistent with this suggestion, the cattle egret, black-crowned night heron, and little egret (the major JEV reservoir) are migratory species in at least the countries of Japan, Korea, and China [28,29].

In summary, this study reports that at least two distinct genotypes of JEV have circulated in South Korea. Genotype 3 strains were predominant in Korea before 1993, when genotype 1 strain K93A07 was first isolated. The two genotypes were detected simultaneously in 1994 but since then, only genotype 1 has been isolated in South Korea.

## Competing interests

The authors declare that they have no competing interests.

## Authors' contributions

SMY performed the experiments and contributed to the preparation of the manuscript. JEC, SYK, JR, and WYC collected the specimens and contributed to the data analysis. YRJ and MGH contributed to the data analysis and the preparation of the manuscript. YEJ designed the study, performed the experiments, and prepared the manuscript. All authors have read and approved the final manuscript.
